# Coping with stillbirth: Insights from parents in rural Limpopo, South Africa

**DOI:** 10.4102/jphia.v16i1.1250

**Published:** 2025-07-08

**Authors:** Lunghile Shivambo, Dumile Gumede

**Affiliations:** 1 Department of Development Studies, College of Human Sciences, University of South Africa, Pretoria, South Africa; 2 Faculty of Health Sciences, Durban University of Technology, Durban, South Africa

**Keywords:** stillbirth, coping mechanisms, parents, rural South Africa, Transactional Model

## Abstract

**Background:**

Stillbirth continues to be a significant global public health issue. Effective coping mechanisms are essential for parents to process their grief and heal after a stillbirth. However, research on how South African parents, particularly in rural areas, cope with stillbirth is limited.

**Aim:**

This study explored the coping mechanisms used by parents following stillbirth, using the Transactional Model.

**Setting:**

The study was conducted in the Mopani District, Limpopo province, South Africa.

**Methods:**

A qualitative exploratory design was employed, involving in-depth interviews with 12 purposively selected parents. Data were collected in the participants’ preferred language, Xitsonga, then transcribed, translated into English and thematically analysed using Atlas.ti.

**Results:**

Emotion-focused coping strategies centred on acceptance, avoidance, reframing the loss, sharing experiences of stillbirth, receiving support from healthcare professionals and seeking prayer and spiritual guidance. In contrast, the problem-focused coping strategy involved distraction through meaningful activities.

**Conclusion:**

Despite employing all these strategies, unresolved grief may still impede effective coping.

**Contribution:**

The study emphasises the significance of multidisciplinary care that integrates psychological, social, and spiritual support to address the complex emotional needs of grieving parents.

## Introduction

Stillbirth, defined as the death of a foetus after 22 completed weeks of gestation,^1^ remains a significant global public health issue. Globally, approximately 2 million stillbirths occur every year,^2^ with sub-Saharan Africa having the highest stillbirth rate in the world. In South Africa, stillbirths are prevalent,^3^ particularly in rural settings, where access to healthcare and mental health support is limited.^4^ Evidence shows that stillbirths are primarily attributed to pregnancy complications, insufficient access to quality healthcare and the absence of essential health services, particularly in low-income and middle-income countries.^2^ Stillbirth, while often seen as a biomedical issue, also represents a profound psychosocial challenge that can have lasting effects on the lives of parents.^5^

Stillbirth is an emotionally devastating experience and the mechanisms parents use to cope with the loss are deeply influenced by a combination of cultural, social and personal factors.^6,7^ The emotional and psychological impact of losing a child during pregnancy can significantly alter the parents’ emotional well-being, relationships and daily functioning. The consequences of stillbirth often leave parents grappling with profound grief and distress.^5^ A systematic review of qualitative studies found that parents who have experienced stillbirth often endure intense grief, guilt, pain and social stigma.^8^

Cultural, societal and individual factors shape how parents process grief and find healing, with social support and coping mechanisms playing key roles in their recovery.^6^ A meta-synthesis revealed that spiritual resources play a critical role in helping parents cope with the loss of a stillborn child.^9^ Through the grieving process, these resources provided parents with a sense of meaning, hope and resilience, enabling them to navigate their emotional pain and find the strength to heal. Spiritual practices, such as prayer and reflection, often acted as a foundation for emotional recovery, fostering connection and comfort during a profoundly challenging time.

However, limited research has explored specific coping mechanisms used by parents in rural South African contexts, leaving gaps in understanding how parents navigate grief in such contexts. Much less is known about the coping strategies used by parents in mitigating these psychosocial consequences of stillbirth and how these align with the theoretical dimensions of stress and coping. This lack of knowledge impedes the development of effective support systems tailored to their needs. Therefore, there is a need to explore the coping mechanisms of parents, who have experienced a stillbirth in the rural South African context.

The Transactional Model of Stress and Coping^10^ provides a relevant theoretical framework for understanding how parents in this context respond to the stressors associated with stillbirth. The model suggests that individuals actively manage stressful situations by assessing and utilising available resources.^10^ It also distinguishes between different coping approaches, such as emotion-focused and problem-focused coping, and provides insight into how these strategies can be applied to navigate stress effectively. It emphasises the dynamic interplay between emotion-focused coping strategies and problem-focused coping strategies, offering a structured lens to explore parental experiences. This model conceptualises stress as a dynamic process that arises from the interaction between an individual and the environment, emphasising the role of personal appraisal and coping strategies in managing stress.^11^ The model conceptualises cognitive appraisal as the process by which individuals assess whether a specific event or experience affects their well-being. Through this process, individuals evaluate the situation and determine the resources available to manage a perceived threat or challenge effectively.^10^ Although the Transactional Model of Stress and Coping has been widely applied in other contexts, its application in understanding stillbirth-related grief in rural South African settings remains sparse. This study fills this gap by employing the framework to examine how parents employ coping strategies in a culturally nuanced way.^10^

This study is particularly important in South Africa with its large population living in lower socio-economic conditions, where existing healthcare services often lack bereavement support following stillbirth.^12^ Understanding parents’ coping strategies can inform relevant interventions, improve healthcare training and guide policymakers in addressing the psychosocial dimensions of stillbirth. Therefore, the purpose of the study was to explore the coping strategies used by parents following stillbirth in rural Limpopo province, South Africa, through the lens of the Transactional Model of Stress and Coping.

## Research design and methods

### Study design

A qualitative exploratory design was employed in this study to gain in-depth insights into the coping strategies parents used following stillbirth. This design was chosen because it allows for a rich understanding of participants’ lived experiences, enabling the exploration of complex emotional, social and cultural factors influencing their coping mechanisms.^13^

### Study setting

The study was conducted in the Mopani District, Limpopo province, South Africa. In this district, cultural traditions and community dynamics significantly influence responses to grief. The study area falls under the jurisdiction of traditional leadership, reflecting the region’s strong adherence to cultural governance and societal norms.

### Population and sampling strategy

The study population consisted of parents (both mothers and fathers) aged 18 years and older, who had experienced stillbirth in a rural village in Limpopo province, South Africa. Purposive and Snowball sampling strategies were employed to select participants, ensuring that those with relevant experiences were included in the study. This combination of Purposive and Snowball sampling is effective for qualitative studies exploring sensitive topics in hard-to-reach populations.

### Data collection procedures

Data collection for this study involved in-depth interviews conducted by the first author. Interviews were carried out in the participants’ preferred language, Xitsonga, to ensure cultural relevance and comfort. An interview guide was used to explore the coping strategies parents employed following stillbirth, allowing for flexibility in responses, while ensuring that key topics were covered. During the interviews, participants were asked a series of questions about how they coped with their loss, including whether they received support from family, friends, religious institutions or healthcare facilities. Additional questions focused on the specific coping strategies they employed, with probes exploring whether they sought counselling or spoke to someone for support. Participants were also invited to share recommendations on how other bereaved parents could navigate the experience of stillbirth. The interviews took place in the participants’ homes between August 2023 and September 2023, providing a familiar and private setting conducive to open dialogue. Each session was audio-recorded to ensure accurate data capture, with interview durations ranging from 30 min to 60 min. This approach allowed for rich, qualitative data collection, while also respecting participants’ time.^14^

### Data analysis

Interviews were transcribed verbatim and translated into English. The study used thematic analyses by following 10 steps and procedures for conducting qualitative data analysis.^15^ The first author coded all interviews, and the second author reviewed a random 30% sample. The Transactional Model guided the coding process. In instances where discrepancies arose between coders, the team engaged in discussions to resolve differences and establish a consensus on the way forward. Both inductive and deductive analysis approaches were employed. The Transactional Model guided the identification of two primary themes: (1) emotion-focused coping strategies and (2) problem-focused coping strategies. Sub-themes were further derived from interview data. To ensure robust thematic analysis, the authorship team reviewed interview transcripts collaboratively. Atlas.ti software facilitated data management and coding.

A realist approach was adopted, where participants’ perspectives were accepted as presented, ensuring authenticity in the interpretation of findings. The study adhered to the Consolidated Criteria for Reporting Qualitative Research (COREQ) guidelines, ensuring methodological rigour and transparency.^16^

### Trustworthiness

To ensure the trustworthiness of the study, four key strategies were employed: credibility, transferability, dependability and confirmability.^17^

Firstly, to ensure credibility, the research team adhered strictly to established qualitative research standards. This involved a continuous and thorough analysis of the data, with repeated listening to audio recordings and a detailed review of the transcripts. Such an approach allowed the researchers to fully immerse in the dataset, thereby enhancing the validity of the findings.^13^

In terms of transferability, the researchers provided a detailed description of the individuals, the setting and the context in which the study was conducted. This enables readers to assess the applicability of the findings to other similar contexts, a crucial aspect of qualitative research.^18^

To ensure dependability, comprehensive records were maintained, including raw data, interview transcripts, field notes and all other relevant documentation. This rigorous documentation process allows for transparency and accountability in the research process, facilitating replication and verification of findings.^19^

Lastly, to ensure confirmability and minimise potential bias, the researchers used direct quotes from participants to support the emerging themes. This strategy ensures that the study’s findings are grounded in participants’ authentic perspectives, reducing the likelihood of researcher bias and enhancing the overall reliability of the results.^13^

By adhering to these strategies, the study aimed to establish and maintain a high level of trustworthiness throughout the research process.

### Ethical considerations

To conduct this study, ethical clearance was obtained from the University of South Africa’s College of Human Sciences’ Research Ethics Committee (No. 64079228_CREC_CHS_2023). Permission to undertake the study in the community was obtained from the Royal Council to enter the community. All procedures performed in the undertaken studies involving human participants were in accordance with the ethical standards of the institutional research committee and with the 1964 Helsinki Declaration and its later amendments or comparable ethical standards were also followed. Written informed consent was obtained from all the individual participants involved in the study.

## Results

### Demographic profile of participants

The study included 12 participants, comprising eight mothers and four fathers. Their ages ranged from 23 years to 61 years, reflecting a diverse demographic spectrum. Participants reported experiencing one or two stillbirths. Among them, all but one had a subsequent live child following their stillbirth experience, highlighting varying family trajectories and contexts within the samples.

### Themes and sub-themes

This section is grounded in the Transactional Model of Stress and Coping, which serves as the conceptual framework. The coping strategies identified are categorised into two primary approaches: emotion-focused and problem-focused coping. These categories were treated as overarching themes from which specific sub-themes were derived based on the study findings. The sub-themes are illustrated with verbatim quotations from participants to provide authenticity and contextual depth. Table 1 presents a summary of the themes and sub-themes, followed by a detailed explanation of the results.

**TABLE 1:** Coping strategies employed by parents following stillbirth.

Themes	Sub-themes
1. Emotion-focused coping strategies	1.1. Acceptance and emotional adjustment 1.2. Avoidance and emotional suppression 1.3. Reframing the loss 1.4. Family support and shared experiences of stillbirth 1.5. Support from healthcare professionals 1.6. Reliance on prayer and spiritual guidance for emotional healing
2. Problem-focused coping strategy	2.1. Distraction through meaningful activities

#### Theme 1: Emotion-focused coping strategies

Six broad sub-themes emerged from the analysis regarding the emotion-focused coping strategies that participants employed in response to experiencing stillbirth. The emerging sub-themes include: acceptance and emotional adjustment; avoidance and emotional suppression; reframing the loss; family support and shared experiences of stillbirth; support from healthcare professionals; and reliance on prayer and spiritual guidance for emotional healing. These strategies focused on managing the emotional response to a stressful situation rather than directly addressing the problem itself.

**Sub-theme 1.1: Acceptance and emotional adjustment:** The findings from this study reveal that the participants demonstrated an emotional processing of the grief and loss as they reflected on accepting the loss of the stillborn babies. Their focus was on managing emotional pain by coming to terms with the reality of the loss rather than addressing or altering the cause. The participants expressed comfort in the fact that they did not have the opportunity to form a deeper bond with the babies:

‘Yes, you know, because I have accepted what happened. These were stillborn babies, so I never saw them alive or had the chance to care for them. I never had the opportunity to raise them, watch them grow or see them run around. I suppose I find some comfort in that, and maybe that’s why I’m able to cope with the loss. I believe that if they had been older, the situation might have been different. As you can see, I have other children with whom I’m familiar. I can talk to them, and they’re very fun to be around. But for those two, they didn’t even make it home alive.’ (P02-F-38 years)

In addition, participants employed positive self-talk as a strategy to help them accept the loss of their stillborn babies, using affirmations and comforting messages to reconcile with the grief they experienced. One participant stated:

‘I practiced self-talk until I came to terms with the fact that I had lost a baby. Over time, I gradually accepted the loss and started to feel better.’ (P08-F-32 years)

These results suggest that the participants found comfort in their acceptance of the situation.

**Sub-theme 1.2: Avoidance and emotional suppression:** The participants described suppressing or pushing away the painful thoughts of their loss, and then, when they dwell on it, they tried to manage the emotional distress by letting it go. Talking about this issue, participants said:

‘No, I haven’t discussed it with anyone. Sometimes, when the thought crosses my mind, I just push it aside. Other times, I think deeply about it, but eventually managed to let it go. However, I always remind myself that in addition to the five children I already have, I would have had five more – four boys and one girl. I can’t help but keep counting.’ (P01-F-57 years)

‘I didn’t talk to anyone about it; I couldn’t bring myself to. Instead, I would speak to myself and write down everything that had happened. I was truly broken, really shattered. I was young and didn’t understand many things, but what I do remember is how deeply hurt I was.’ (P04-F-39 years)

‘No, we hardly talk about it here at home. I can tell that most people are still hurting, so I suppose we avoid discussing it because we don’t want to think about it.’ (P11-M-24 years)

Overall, these results indicate that the participants coped with the grief and loss by diverting attention away from the painful thoughts and attempting to suppress them, while also focusing on the positive aspects of their current family situation.

**Sub-theme 1.3: Reframing the loss:** The participants expressed finding comfort or solace through emotional regulation and reframing their experience of loss rather than actively trying to change or solve the circumstances of the loss. One participant stated:

‘I am coping because, despite the loss of some children, I still have others who are alive and for whom I continue to live. Additionally, they now have children of their own, so I feel that the legacy I’ve left is now bearing fruit, filling the gap. I always find comfort in the thought that, although I lost some children, I still have others.’ (P07-M-61 years)

Another participant added a sense of fulfilment to the role of becoming a mother again:

‘I only started to feel better when I had my second child. It was then that I felt I could be a mother again and begin to socialise with others.’ (P08-F-32 years)

The participants reinterpreted their experience of loss by focusing on the positive outcomes of having living children after a stillbirth. While some participants found consolation in the positive outcomes of having living children after a stillbirth, others continued to experience unresolved grief and emotional pain, even with the presence of a surviving child. For example, one participant said:

‘I was completely torn apart, overwhelmed by so much pain, and I wasn’t okay. After losing my child, I felt compelled to conceive and have more children, as if it would somehow take revenge or fill the void left by that loss. Yes, I did have other children, but they didn’t fill that gap. It especially hurts when I see the women I used to go to the clinic with, holding their babies – now those children are all grown. It truly pains me, and I won’t lie, I still feel that pain when I think about it. When I gave birth to my daughter, I hoped it would close that gap, but it hasn’t. I still think about him.’ (P05-F-51 years)

What emerges from the results reported here is that although the participants had subsequent children after experiencing stillbirths, the emotional pain of their earlier loss remained unresolved, suggesting that the birth of living children did not fully ease the grief they continued to carry.

**Sub-theme 1.4: Family support and shared experiences of stillbirth:** The participants mentioned that they found emotional strength through the support of family members, whose similar experiences of stillbirth provided a sense of solidarity and reassurance, contributing to their emotional healing. As one participant put it:

‘My family continuously encouraged me to accept what had happened, and they shared their own experiences of losing babies, much like I had. My mother told me that she also had a stillborn baby and had managed to accept the loss. Hearing this, I reassured myself that I was not the only person to have experienced stillbirth. It showed me that such things happen and could happen to anyone. If my mother had gone through it and survived, then I, too, could accept the loss and move on. My grandmother also shared that she had experienced a stillbirth, and it was from their stories that I drew strength. Their experiences taught me that these things happen, and I had to accept the loss as part of life. This is how I found the strength to cope and began to heal.’ (P02-F-38 years)

In summary, these results show that the shared experiences of stillbirth with family members helped the participants emotionally process the loss, enabling them to accept that stillbirth was a common, painful experience and that others in their families have survived and moved forward.

**Sub-theme 1.5: Support from healthcare professionals:** The participants reflected on the support they received from healthcare professionals, including nurses and a social worker, who reassured them that stillbirth is a common experience, and that acceptance is part of the healing process. For instance, the following excerpts demonstrate the support from healthcare professionals as a coping mechanism:

‘I think about six nurses came to me to offer counselling. They told me that I needed to accept what had happened, explaining that this is something that usually occurs, so I must accept it. I then told myself that, indeed, this kind of thing does happen. I had heard other women speak about losing their babies, but it had never happened to me before. However, I now realize that it does happen.’ (P06-F-47 years)

‘The nurses referred me to another social worker; I can’t remember her name because this happened a long time ago. My counselling session with her was very helpful. She told me that I wasn’t the first person to experience a stillbirth and that with time, I needed to accept it.’ (P05-F-51 years)

In summary, for the participants in this study, the understanding that stillbirth happened to others also facilitated the healing process for the participants to come to terms with their loss.

**Sub-theme 1.6: Reliance on prayer and spiritual guidance for emotional healing:** The participants found consolation in religious practices (e.g. praying and church attendance) and spiritual guidance from others in their church community:

‘God has been my source of strength. I honestly don’t know how I would have survived all of this, but as you can see, I am strong and doing well, all thanks to His grace. I enjoy being around other congregants at church because it gives me the chance to talk about anything, and it helps me forget my problems.’ (P01-F-57 years)

‘Going to church to pray helped me a lot.’ (P11-M-24 years)

‘Elderly women from my church sat down with me and offered words of encouragement. They even read from the Bible, quoting scriptures. After that, I accepted the situation and moved on with my life. Not long after, I became pregnant again, and this time, the baby survived.’ (P02-F-38 years)

These results suggest that the religious activities helped the participants process their grief and find meaning, allowing them to cope with the emotional consequences of stillbirth.

#### Theme 2: Problem-focused coping strategies

From the analysis of the problem-focused coping strategies used by participants following a stillbirth, only one sub-theme emerged: distraction through meaningful activities. This sub-theme highlights the proactive measures participants took to manage their stressful situation, focusing on engaging in purposeful tasks to mitigate emotional distress.

**Sub-theme 2.1: Distraction through meaningful activities:** Participants engaged in meaningful activities that not only provided a sense of purpose but also served as a distraction from grief or negative emotions associated with their loss, as shown in the excerpts:

‘At times, I keep myself occupied with household chores, as they help distract me and ease my mind from dwelling on many thoughts.’ (P01-F-57 years)

‘I run a firewood-selling business, which keeps me busy and helps distract me from overthinking. I am also a coach, helping young boys who are interested in soccer. It benefits me because it occupies my time. Even now, after talking to you, I’m heading there. Sometimes, when I think about it at night, I find myself looking forward to the next day, so I can be with the boys, training them on the soccer field [*laughs*].’ (P07-M-61 years)

Overall, these results indicate that the participants engaged in external activities to cope with emotional distress.

## Discussion

This study explored the coping strategies employed by parents in rural Limpopo, South Africa, after experiencing stillbirth, using the Transactional Model of Stress and Coping^10^ as a guiding framework. The findings reveal that participants’ reliance on both emotion-focused and problem-focused coping strategies reflects their efforts to manage the emotional impact and practical consequences of stillbirth. Despite these coping strategies, unresolved grief may still hinder effective coping, with some parents continuing to experience emotional pain despite utilising various coping strategies.

The results demonstrated that participants predominantly utilised emotion-focused coping strategies to address their grief. These strategies, including acceptance and emotional adjustment, avoidance and emotional suppression, reframing the loss, family support, spiritual guidance and healthcare support, aimed to regulate their emotional responses rather than solve the stressor itself.

Participants highlighted the importance of acceptance in processing their loss. The participants’ belief in the unchangeable nature of their circumstances contributed to their acceptance of the loss they experienced because of the stillbirth. This coping mechanism aligns with research suggesting that acceptance facilitates emotional healing by enabling individuals to reconcile with reality.^20^ In another study, acceptance and normalisation were used by participants as emotional regulation mechanisms to deal with distressing circumstances.^21^ In addition, positive self-talk, as reported by participants, also highlights the effectiveness of cognitive reframing in coping with grief.

Some participants described efforts to suppress painful memories and redirect their focus away from grief. These participants seem to avoid confronting or discussing the pain of loss, either by pushing thoughts aside, internalising their feelings, or not engaging with others about the grief. This behaviour highlights the difficulty of confronting the emotional pain of loss and the strategies individuals use to protect themselves from further emotional distress. The literature on avoidance coping presents mixed findings. Some studies suggest that avoidance coping strategies, such as emotional suppression or denial, can provide short-term relief from overwhelming grief or distress.^22^ However, other research indicates that prolonged reliance on avoidance can hinder long-term adjustment and emotional healing, contributing to the development of complications like prolonged grief disorder.^23^ Daros and Williams^22^ suggest that avoidance coping can function as a short-term strategy to alleviate emotional distress following traumatic events such as grief. While this approach may provide immediate relief, it does not necessarily address the underlying emotional issues, potentially delaying long-term healing. Folkman and Moskowitz^24^ further emphasise that although avoidance can offer temporary comfort, its prolonged use may hinder emotional recovery and lead to negative psychological outcomes, including prolonged grief or maladaptive coping mechanisms. This highlights the tension between short-term emotional relief and the risk of long-term psychological consequences when avoidance is used as a predominant coping strategy.

Reframing their loss by focusing on surviving children or future pregnancies allowed participants to derive meaning from their experiences. The findings highlight a significant contrast between participants who managed to reframe their grief by focusing on the positive outcomes of subsequent births, and those who continued to grapple with grief despite the presence of living children. This contrast highlights the variability of coping strategies, even under similar circumstances, reflecting the individual nature of grief.^24^ Such differences emphasise the need for tailored support, as personal, cultural and situational factors influence emotional responses to loss. The complexity of these emotional journeys calls for a nuanced understanding of how grief is processed and how support systems can be adapted to individual needs. Furthermore, family support, particularly shared narratives of loss, was pivotal in reinforcing resilience. These findings show that family solidarity fosters emotional adjustment by normalising loss and providing collective strength.^25^

Religious practices, including prayer and church attendance, were central to emotional recovery. Participants reported that spiritual guidance provided comfort and helped contextualise their experiences within broader existential beliefs. This reflects the findings of previous studies, which emphasise the role of spirituality in enhancing psychological well-being among bereaved individuals in African contexts^26^, and religious coping is an important strategy in helping to cope with stressful situations.^21^ Our results are also consistent with previous studies in that the practice of religious activities provided means to alleviate suffering and facilitated acceptance of the loss.^8,27^ However, there may be a need to foster collaboration among psychologists, pastoral counsellors and other relevant professionals to combine evidence-based psychological interventions with spiritual guidance. This multidisciplinary approach can offer a more comprehensive strategy for addressing grief, addressing both emotional and spiritual needs in bereavement care.

Participants acknowledged the value of counselling from healthcare workers, which provided reassurance and normalisation of their loss. A previous study highlighted the importance of empathetic healthcare support in promoting coping and reducing emotional isolation.^28^ This shows how useful the support from healthcare workers is in helping parents following stillbirth to deal with the stressful situations they encounter. Similar findings have been reported in previous studies showing that healthcare workers play a vital role in supporting and helping distressed individuals.^21^ To support parents following a stillbirth, healthcare professionals can implement interventions that focus on empathetic care, compassionate communication and psychological support.

The study also identified a single problem-focused coping strategy: engaging in meaningful activities. This approach reflects participants’ efforts to distract themselves from grief through purposeful engagement, such as household chores, business endeavours or community activities. Research evidence supports the effectiveness of such strategies in fostering resilience by creating a sense of achievement and routine.^29^ This aligns with findings from cultural contexts where collective activities help individuals integrate grief into daily life.^27^

However, problem-focused coping strategies like engaging in purposeful activities may help distract individuals from immediate pain, but might not address deeper emotional grief effectively. This could result in unresolved grief, which might resurface in other aspects of their lives.^30^ The findings of the study indicate that while some participants found consolation in the positive outcomes of having living children after a stillbirth, others continued to experience unresolved grief and emotional pain, even with the presence of a surviving child. In another study, self-distraction was used as a coping mechanism by the participants to distract themselves from thoughts about the stressful condition.^21^ Therefore, while distraction can be helpful, it may also act as a form of avoidance, delaying the emotional processing of the loss. Avoidance has been linked to prolonged grief symptoms in bereavement studies.^23,31^ A psychometric validation study indicates that individuals who continue to rely on avoidance strategies for coping with grief beyond 6 months to 12 months after a traumatic event are at an increased risk of developing symptoms associated with prolonged grief disorder in the future.^23^ Anderson et al.^27^ suggest that when bereaved parents focus primarily on the negative emotions associated with grief, they often struggle to move past these feelings and experience difficulties in the grieving process. This focus on negative emotions can create barriers to emotional healing and adjustment, making it challenging for parents to transition towards more adaptive coping mechanisms.

To mitigate the risks associated with avoidance and self-distraction, evidence-based coping strategies should be encouraged. Educating individuals about a range of coping strategies can enhance their ability to engage with both problem-focused and emotion-focused coping approaches, fostering greater resilience and adaptability in navigating grief. By equipping individuals with diverse tools to address emotional and practical challenges, such psychoeducation can support more comprehensive and effective grief management. Moreover, developing a sense of meaning after stillbirth is crucial for long-term psychological well-being. In this study, parents reframed their loss by focusing on surviving children or future pregnancies. It is reported that parents who reframe their experience and find meaning in their loss often experience better emotional outcomes.^32^

### Strengths, limitations and future research

This study employed a qualitative design, allowing for rich, detailed insights into the personal experiences of parents coping with stillbirth in rural Limpopo province, South Africa. By using in-depth interviews, the study captured the emotional and psychological dimensions of the participants’ experiences, which would be difficult to assess through quantitative measures alone.^33^ Additionally, by applying the Transactional Model of Stress and Coping,^10^ the study provides a theoretical framework for understanding how individuals appraise and cope with grief, contributing to the conceptualisation of emotional responses to stillbirths.

While the study provides valuable insights into the experiences of a specific group of parents in rural Limpopo, the relatively small sample size may limit the generalisability of the findings to other regions or populations. Moreover, although the study includes both male and female participants, the perspectives of male parents on stillbirths and the grief they experienced were underrepresented.

Future research should investigate the different ways men and women experience grief after stillbirth, as well as how gender roles and cultural expectations impact coping strategies. Longitudinal research would provide valuable insights into how grief evolves and the effectiveness of various coping mechanisms in facilitating long-term recovery. Additionally, intervention-based research could help determine the most effective strategies for helping parents in coping with stillbirth. Finally, future studies should consider employing diverse theoretical models to enhance the understanding of coping mechanisms following stillbirth.

## Conclusion

The coping strategies identified in this study align with the Transactional Model of Stress and Coping, demonstrating that participants employed a range of strategies to mitigate the emotional and practical impacts of stillbirth. The reliance on emotion-focused coping strategies emphasises the role of family, spirituality and supportive networks in facilitating emotional recovery, while the problem-focused coping strategy illustrates the significance of purposeful engagement in daily activities. Although various coping strategies were employed, unresolved grief can obstruct parents’ ability to fully recover from the trauma of stillbirth. Such unresolved emotions may remain latent, potentially manifesting later in other areas of their lives, affecting emotional well-being and interpersonal relationships. Providing psychoeducation on diverse coping strategies might empower individuals to explore both, problem-focused and emotion-focused mechanisms, improving overall adaptability in managing grief. Our findings contribute to a nuanced understanding of bereavement coping mechanisms in rural Mopani District, Limpopo province, South Africa, and provide a basis for culturally informed interventions to support parents experiencing stillbirth. Future research should aim to address its limitations by expanding sample size, exploring gendered experiences and conducting longitudinal studies. By doing so, future studies can further enrich our understanding of how parents navigate grief and offer guidance for improving support and interventions.
